# Synbiotics Alleviate the Gut Indole Load and Dysbiosis in Chronic Kidney Disease

**DOI:** 10.3390/cells10010114

**Published:** 2021-01-09

**Authors:** Chih-Yu Yang, Ting-Wen Chen, Wan-Lun Lu, Shih-Shin Liang, Hsien-Da Huang, Ching-Ping Tseng, Der-Cherng Tarng

**Affiliations:** 1 Institute of Clinical Medicine, School of Medicine, National Yang-Ming University, Taipei 11221, Taiwan; cyyang3@vghtpe.gov.tw; 2 Stem Cell Research Center, National Yang-Ming University, Taipei 11221, Taiwan; 3 Division of Nephrology, Department of Medicine, Taipei Veterans General Hospital, Taipei 11217, Taiwan; 4 Center for Intelligent Drug Systems and Smart Bio-Devices (IDS2B), Hsinchu 30010, Taiwan; 5 Department of Biological Science and Technology, National Chiao Tung University, Hsinchu 30010, Taiwan; afratw@gmail.com (T.-W.C.); wanlun106@gmail.com (W.-L.L.); hsienda.huang@gmail.com (H.-D.H.); 6 Institute of Bioinformatics and Systems Biology, National Chiao Tung University, Hsinchu 30010, Taiwan; 7 Department of Biotechnology, College of Life Science, Kaohsiung Medical University, Kaohsiung 80708, Taiwan; liang0615@kmu.edu.tw; 8 Institute of Biomedical Science, College of Science, National Sun Yat-Sen University, Kaohsiung 80424, Taiwan; 9 Department and Institute of Physiology, School of Medicine, National Yang-Ming University, Taipei 11221, Taiwan

**Keywords:** synbiotics, gut indole, dysbiosis, chronic kidney disease

## Abstract

Chronic kidney disease (CKD) has long been known to cause significant digestive tract pathology. Of note, indoxyl sulfate is a gut microbe-derived uremic toxin that accumulates in CKD patients. Nevertheless, the relationship between gut microbiota, fecal indole content, and blood indoxyl sulfate level remains unknown. In our study, we established an adenine-induced CKD rat model, which recapitulates human CKD-related gut dysbiosis. Synbiotic treatment in CKD rats showed a significant reduction in both the indole-producing bacterium *Clostridium* and fecal indole amount. Furthermore, gut microbiota diversity was reduced in CKD rats but was restored after synbiotic treatment. Intriguingly, in our end-stage kidney disease (ESKD) patients, the abundance of indole-producing bacteria, *Bacteroides*, *Prevotella*, and *Clostridium*, is similar to that of healthy controls. Consistently, the fecal indole tends to be higher in the ESKD patients, but the difference did not achieve statistical significance. However, the blood level of indoxyl sulfate was significantly higher than that of healthy controls, implicating that under an equivalent indole production rate, the impaired renal excretion contributes to the accumulation of this notorious uremic toxin. On the other hand, we did identify two short-chain fatty acid-producing bacteria, *Faecalibacterium* and *Roseburia*, were reduced in ESKD patients as compared to the healthy controls. This may contribute to gut dysbiosis. We also identified that three genera Fusobacterium, Shewanella, and Erwinia, in the ESKD patients but not in the healthy controls. Building up gut symbiosis to treat CKD is a novel concept, but once proved effective, it will provide an additional treatment strategy for CKD patients.

## 1. Introduction

Chronic kidney disease (CKD) affects around 12% of adults worldwide [1], and the prevalence of CKD is increasing year by year due to the habit change of diet and lifestyle. CKD has long been known to cause significant gastrointestinal pathology. CKD can result in profound changes in the microbial ecosystem, microbiome composition, and the function and structure of the digestive tract [2,3,4]. An increase in bacteria that produce indole-forming enzymes and the depletion of bacteria that possess short-chain fatty acid (SCFA)-forming enzymes can lead to gut dysbiosis in human and animal models [5,6]. Of note, the indole in the gut is produced by gut bacteria through the proteolysis of tryptophan. The indole was absorbed from gut villi and entered the portal venous system, followed by hepatic sulfation. We previously reported that indoxyl sulfate enters the circulation via gut villus absorption and is harmful to cardiovascular health in the CKD population [7,8].

Indole-producing bacteria in the digestive tract consume tryptophan, thus serve as the source of the indoxyl sulfate, a gut bacteria-derived uremic toxin [9]. We took advantage of high-throughput next-generation sequencing (NGS) to detect hundreds to thousands of microbiota simultaneously and offer comprehensive culture-free techniques for surveying human microbiome composition and biomolecular activity at the transcriptional level [10,11,12,13]. Building up gut symbiosis to treat CKD is a novel concept [14], but once proven effective will provide an additional treatment strategy for CKD patients. Previous studies have shown that synbiotics could reduce circulating levels of p-cresol in healthy volunteers and hemodialysis patients [15,16,17,18], yet its effects on the gut microbiome and renal outcomes were unknown.

We hypothesized that the indole production availability by intestinal bacteria was a pathogenic factor for CKD. It is crucial to verify the role of intestinal microbiota in indole production for preventing CKD progression. In this article, we examined gut dysbiosis and indole metabolism in CKD rats and end-stage kidney disease (ESKD) patients. An interventional study in CKD rats was also performed using synbiotics to alter the gut microbiota and to investigate whether renal outcomes can be improved.

## 2. Materials and Methods

### 2.1. Human Blood Metabolite Analysis

The Institutional Review Board of the institute approved all protocols before the study began (Approval date: 8 February 2018; IRB-TPEVGH No.: 2016-12-004A#1), and the protocols conformed to the ethical guidelines of the Helsinki declaration. The signed informed consent was obtained from each participant. The human plasma samples were inventoried and immediately stored at −80 °C until processing. Plasma samples were pretreated with 50 μL human plasma and 1400 μL methanol (MeOH; Merck, Seelze, Germany) to precipitate proteins. The individual mixture samples were shaken by vortex for 5 min and followed by centrifugation at 13,400× *g* for 20 min at 4 °C. Individual plasma samples were collected and transferred supernatant into the other tubes and volatilized using a spin vacuum instrument. The lyophilized samples were redissolved with 190 μL 30% acetonitrile (MeCN; Merck, Seelze, Germany) and 10 μL 2 ppm d4-indoxyl sulfate (internal standard). Finally, the redissolved mixture was filtered by a 0.22 μm PVDF filter. Each filtrate was injected into an HPLC-tandem MS system (HPLC-MS/MS, high performance coupled with tandem mass spectrometer; Thermo Finnigan TSQ Quantum Ultra Mass Spectrometer, Thermo Fisher Scientific Inc., Waltham, MA, USA).

The indoxyl sulfate and *p*-cresol sulfate of patients’ plasma samples were detected by HPLC-MS/MS. The MS system was equipped with a high voltage (2.5 kV) Micro ESI ion source. The analytical system was an Acella 1250 UHPLC system (Thermo Fisher Scientific Inc.). The filtrate of *p*-cresol sulfate (APExBIO, Houston, TX, USA), indoxyl sulfate (Sigma-Aldrich, Saint Louis, MO, USA), and d4-indoxyl sulfate (Sigma-Aldrich, Saint Louis, MO, USA) were sequentially injected into the HPLC analytical system via the Acella 1250 autosampler and ultra-high-performance liquid chromatography (UHPLC, Thermo Fisher Scientific Inc., Waltham, MA, USA). The separated column was using a Shiseido HPLC CAPCELL PAK C18 MGII column (150 mm × 1.5 mm, 3.0 μm, Tokyo, Japan). The mobile phases were composed of (A) 0.1% (*v*/*v*) FA in water, and (B) 0.1% (*v*/*v*) FA in ACN, with a 250 mL/min flow rate, and the linear gradient was set as follows: 30% (B) in 2 min, 30–60% (B) in 6 min, 60–98% (B) in 3 min, 98% (B) in 2 min, 98–30% (B) in 0.1 min and 30% (B) in 6.9 min. The calibration curves for quantification of indoxyl sulfate and p-cresyl sulfate were prepared stock solution concentration from 10 ppb to 5000 ppb. And the values of determination of coefficient were 0.9996 of indoxyl sulfate and 0.9987 of *p*-cresol sulfate, respectively. Intra- and interassay coefficients of variation of indoxyl sulfate in serum were all within 10%.

The detection method of the MS/MS was set up with a negative applied voltage of -2.5 kV, and the vaporizing and capillary temperatures were set at 300 °C and 350 °C, respectively. The sheath gas and aux gas flow rate was set at 35 and 10, respectively, with a collision pressure of 1.5 and collision energy adjusted to 22 V. The detection mode of multiple reaction monitoring (MRM) was set MRM transitions 187 > 80 and 187 > 107 belonging to *p*-cresol sulfate, 212 > 80 and 212 > 132 belonging to indoxyl sulfate, and 216 > 84 and 216 > 136 belonging to d4-indoxyl sulfate for quantification. The controlling software, Xcalibur (version 2.2, Thermo-Finnigan Inc., San Jose, CA, USA), was used to acquire the MS spectra and control the mass spectrometer.

### 2.2. Human Fecal Microbiota Analysis

We characterized the microbial communities in the fecal samples of 40 CKD and 22 age gender-matched health controls (Table 1) by 16S rRNA sequencing. Total DNA was extracted from stool samples, and 16S rRNA were amplified and sequenced with Illumina sequencers. We used QIIME [19] for quality filtering, operational taxonomic unit (OTU) picking, and taxonomic assignment. All these OTUs were assigned to their genus-level, and we compared the abundance of each genus between healthy controls and CKD patients.

### 2.3. The CKD Animal Model

All animal experiments were performed following the guidelines of the Institutional Committee for Animal Experimentation of National Chiao Tung University (Approval date: 30 January 2018; No. NCTU-IACUC-107001). The model of adenine-induced CKD in rats is well established and is similar to CKD in humans [20,21,22]; therefore, the adenine diet model was used in this study. Male Sprague–Dawley (SD) rats aged 9–10 weeks (*n* = 36, body weight (BW) 375 ± 13 g, *n* = 6 for each subgroup) were randomly divided into two models, six experimental groups. Male SD rats were purchased from BioLASCO Taiwan Co., Ltd. and were housed with two rats per cage at the Laboratory Animal Center, National Chiao Tung University. The animals were fed with sterilized water and food. The bred environment was well-monitored and controlled (12-h light/dark cycle, 22 ± 2 °C, and 62 ± 5% humidity). The BW of animals was measured every week. The blood was collected for biochemical analysis every two weeks.

### 2.4. The Animal Model 1: Concomitant Five-Week Adenine Diet and Five-Week Synbiotic Treatment

**Figure 1:**
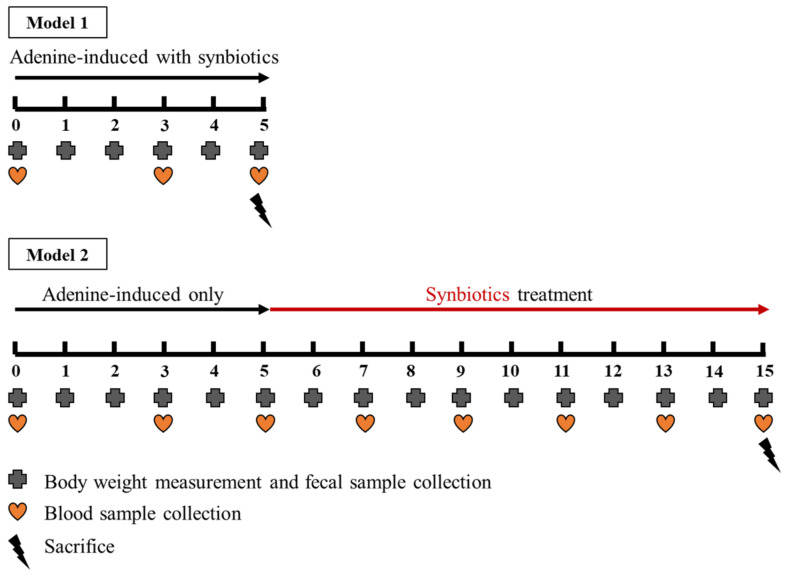
Adenine-induced CKD rat model.

Both prebiotic and probiotic have been found that can modulate intestinal bacterial growth and affect gut microbiota [23,24]. The synbiotics supplementation was further examined in adenine-induced CKD rats. To avoid the variable effect of adenine, we measured the BW of rats and administered the calculated amount of adenine accurately via oral gavage [25,26,27]. As shown in Figure 1, three experimental groups were control rats, adenine-induced CKD rats oral gavage with adenine 250 mg/kg of BW/day (99% pure, Alfa Aesar, Ward Hill, MA, USA), and synbiotics-treated CKD rats (oral gavage with concomitant adenine 250 mg/kg BW/day and synbiotic treatment 10^9^ CFU of probiotics combination of *Lactobacillus* sp., *Bifidobacterium* sp., and *Streptococcus* sp. [16,24,28,29] with the equal bacterial numbers, and inulin (1 g/kg BW/day; Chicory, Sigma-Aldrich, MO, USA) as prebiotic supplementation [17,30,31] up to five weeks). The probiotic strains (*Lactobacillus* sp., *Bifidobacterium* sp., *Streptococcus* sp.), were provided by Glac Biotech Co., Ltd., Tainan, Taiwan.

### 2.5. The Animal Model 2: Five-Week Adenine Diet Followed by 10-Week Synbiotic Treatment

Three experimental groups were control rats, adenine-induced CKD rats (oral gavage with adenine 250 mg/kg BW/day for five weeks then fed with the regular chow diet), and synbiotics-treated CKD rats (oral gavage with adenine 250 mg/kg BW/day for five weeks and then fed with regular chow diet and oral gavage with synbiotics 10^9^ CFU of probiotics combination with the equal amount of *Lactobacillus* sp., *Bifidobacterium* sp., *Streptococcus* sp., and inulin (1 g/kg BW/day) as prebiotic supplementation in the following 10 weeks).

### 2.6. Rat Serum Biochemistry and Kidney Pathology

The serial kidney function was evaluated by serum biochemistry of blood urea nitrogen (BUN) and creatinine. Blood samples were collected into 1000 μL from the tail vein of the rats. After the blood samples stayed at 4 °C for 30 min to 1 h, centrifugation was performed at 3000 rpm for 30 min at 4 °C to obtain serum for biochemical analysis. At sacrifice, the blood was collected, and the kidneys were fixed in 10% buffered formalin. Fixed kidney tissues were trimmed, dehydrated with ethanol, embedded in paraffin, and thin sections of kidneys were cut onto glass slides as previously reported [21]. Routine histological stains with hematoxylin and eosin stain (H and E) were conducted for general histology examination. Masson’s trichrome stains were used to assess collagen deposition.

### 2.7. Rat Fecal Microbiota Analysis

We characterized the microbial communities in the fecal samples of healthy control at weeks 0 and 8 by 16S rRNA sequencing. Total DNA was extracted from stool samples, and 16S rRNA were amplified and sequenced with Illumina sequencers. All these OTUs were assigned to their genus-level, and we compared the abundance of each genus between healthy controls, CKD, and CKD + synbiotics rats.

### 2.8. Kovács Analysis for Rat and Human Fecal Indole Quantification

The Kovács analysis is the most widely used method for detecting indole-producing bacteria. The Kovács analysis was based on a previous publication [32] and modified using 100 μL of the above-described indole standards in 70% ethanol or samples of unknown indole concentrations. The samples were incubated with 150 μL of Kovács reagent (Sigma-Aldrich, MO, USA) for up to 30 min at room temperature. The reaction produced a soluble product, which was analyzed spectrophotometrically at 530 nm using a Hitachi U-3900 spectrophotometer (Hitachi High-Tech, Schaumburg, IL, USA).

### 2.9. Statistical Analysis

Statistical analysis was performed using GraphPad Prism 5 software (GraphPad Software, CA, USA). Graphs represented the means ± SEM. Chi-square analysis or Fisher’s exact test was used for comparison of categorical variables as appropriate. Continuous variables were checked for normality of distribution before Student’s *t*-test and were compared by Pearson correlation, Student’s *t*-test, or one-way analysis of variance (ANOVA) followed by Tukey’s tests for each pair of multiple comparisons, as appropriate. All probabilities are two-tailed, and a *p*-value of less than 0.05 is considered to be statistically significant.

## 3. Results

### 3.1. Human Samples

#### 3.1.1. Demographic Characteristics of Human Study Participants

We enrolled 62 study subjects, among which 40 patients were on hemodialysis while the other 22 were healthy controls. We examined and analyzed their gut microbiome and fecal indole amount. Compared to controls (*n* = 22), whose renal function was normal, we found a positive correlation between the gut microbiome and renal function in the observational study in ESKD patients. The demographic data were listed as follows (Table 1).

#### 3.1.2. Characteristics of the 16s Microbiome in ESKD Patients

As shown in Table 2, we compared the gut microbiota between ESKD participants and healthy controls. The bioinformatic results of human gut microbiota showed that *Streptococcus*, *Alistipes*, *Dorea*, *Parabacteroides*, and *Succinispira* were increased in ESKD patients. As compared to the healthy controls, two SCFA-producing bacteria, *Faecalibacterium* and *Roseburia*, were reduced ESKD patients, which may contribute to gut dysbiosis.

**Figure 2:**
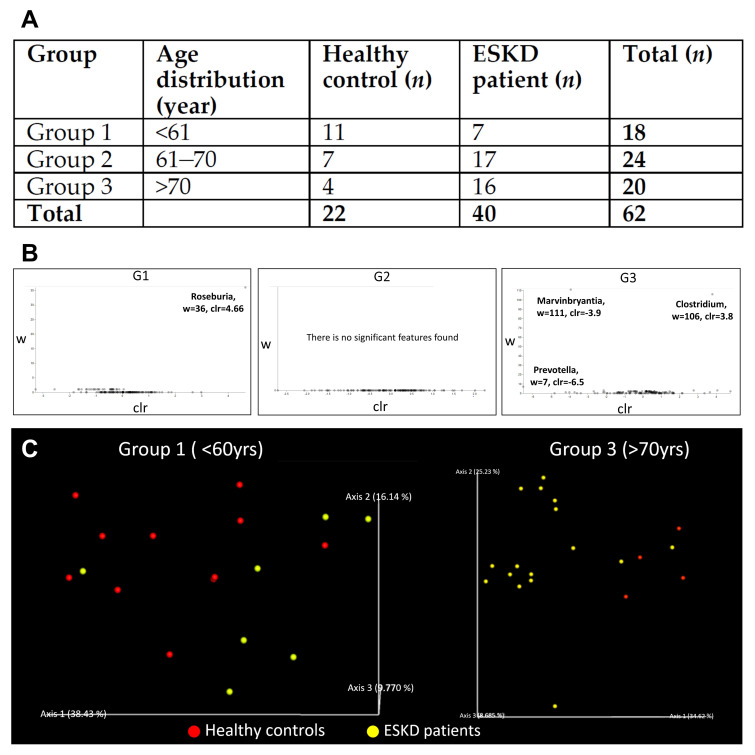
(**A**) Descriptive characteristics of the study population. (**B**) Differential abundance analysis of microbial at the genus level. (**C**) Multidimensional scaling ordination plot of the fecal bacterial community. The overall bacterial compositions can be used to separate most ESKD patients and healthy controls into two groups. (G1) Most ESKD patients were separated from healthy controls with only one exception. (G3) In the elder group, ESKD patients were separated from healthy controls. UniFrac distance metric was used to calculate the pairwise distances between samples. Healthy controls and ESKD patients were shown in red and yellow, respectively.

We believe other clinical factors, such as age, gender, or CKD stage, could also contribute to different microbiome compositions in ESKD patients. However, all CKD patients in our study were at the end-stage. Therefore, we stratified our samples into three subgroups according to their ages. As shown in Figure 2A, there were three age groups denoted by G1, G2, and G3, respectively. The symbol G1 denotes age under 61 years old, and the symbol G2 denotes 61–70 years old, and symbol G3 denotes above 70 years old. Using the multidimensional scaling method for UniFrac phylogenetic distance, which measures the evolutionary distance between microbiota, some specific gut microbiota seemed to present only in ESKD patients. The differential abundance analysis of microbial at the genus level is shown in Figure 2B. We found overall composition profiles were different in group 1 (<61 years) and group 3 (>70 years) (Figure 2C). Further analysis shows that the differences in group 1 were mainly caused by a decrease of *Roseburia* and *Bacteroides* in ESKD patients, while in group 3, the decrease of *Marvinbryantia* and *Prevotella* in ESKD patients was responsible for the differences.

**Figure 3:**
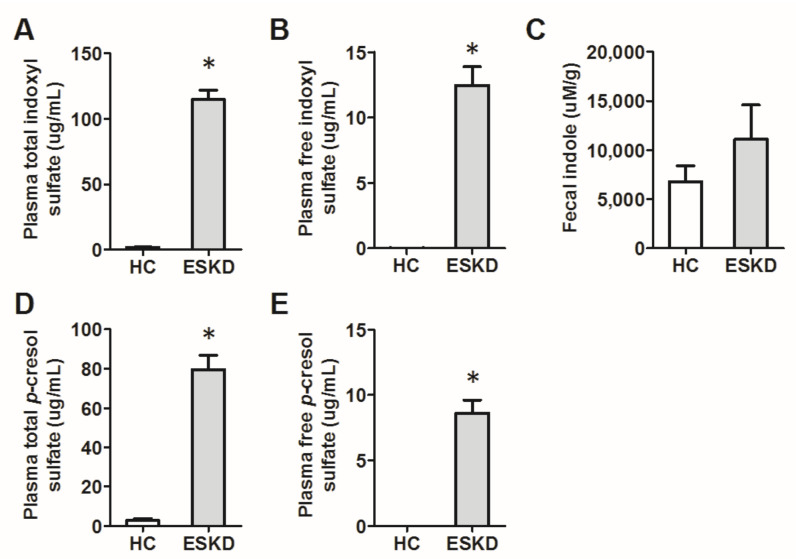
The plasma levels of indoxyl sulfate and *p*-cresol sulfate of end-stage kidney disease (ESKD) patients are significantly higher than healthy controls (HC) (panels (**A**,**B**,**D**,**E**)). The fecal level of a microbial metabolite, indole, tends to be higher in ESKD patients, but the difference did not achieve statistical significance (panel (**C**)). * *p* < 0.05.

Next, in our cohort, we compared blood levels of gut microbe-derived uremic toxins, including indoxyl sulfate and *p*-cresol sulfate, in free and total forms, respectively. We found that their levels are significantly higher in ESKD patients as compared to the healthy controls. The fecal indole tends to be higher in the ESKD patients, but the difference did not achieve statistical significance (Figure 3).

**Figure 4:**
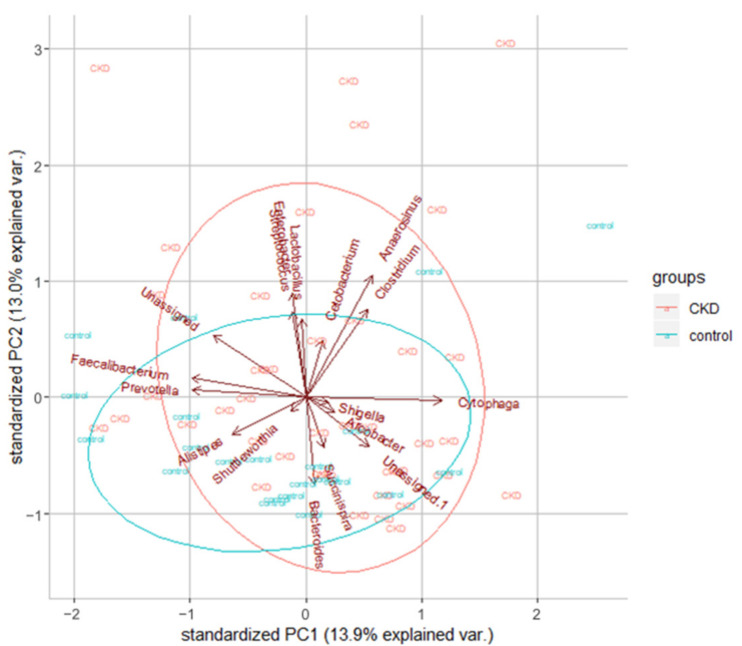
Dimension reduction analysis reveals the overall bacterial compositions slightly different between healthy controls and ESKD patients. The top 20 most abundant microbial genera were used in this analysis and shown with arrows in the plot. Red and green ellipses were plotted according to the 40 ESKD patients and the 22 healthy controls, respectively.

Additionally, we found that the most abundant phyla were *Bacteroidetes*, *Firmicutes*, *Actinobacteria,* and *Proteobacteria* in all samples, which were consistent with previous findings [33]. For all the OTUs, we tried to identify their genus and compare the microbiome between CKD patients and healthy controls. We found several interesting differences. First of all, the most predominant phylum was *Bacteroidetes* for both groups. Second, three genera Fusobacterium, Shewanella, and Erwinia, were only found in the ESKD group but not in the healthy controls. As shown in Figure 4, the global microbiota profile shows a difference between these two groups.

### 3.2. The Animal Model 1: Concomitant Five-Week Adenine Diet and Five-Week Synbiotic Treatment

**Figure 5:**
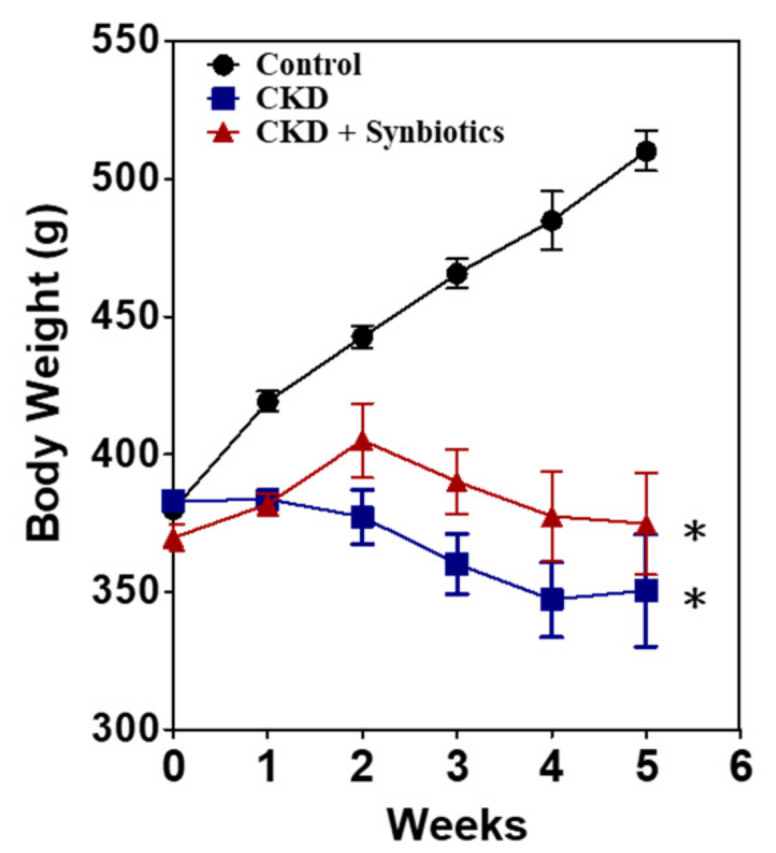
The body weight changes in animal model 1. The bodyweight of the controls (●), CKD rats (■), and synbiotics-treated CKD rats (▲). A significant difference between the controls vs. CKD group vs. synbiotics-treated CKD group was analyzed using ANOVA. *, *p* < 0.05. *n* = 6 for each group.

CKD rats (oral gavage with adenine 250 mg/kg BW/day) lost about 22% and 31% BW compared with the control rats in the 3rd and 5th weeks, respectively. Synbiotics-treated CKD rats only lost about 16% and 22% BW compared with the control rats at the 3rd and 5th weeks, respectively (Figure 5). The kidney function was evaluated by BUN and serum creatinine. At the end of the 3rd and 5th weeks, compared with the control group, the levels of BUN were significantly increased to 108.5 ± 0.9 (7.8-fold) and 214.0 ± 20.0 (13.3-fold) mg/dL in the CKD group (Table 3). The serum creatinine of the CKD group was significantly elevated to 2.8 ± 0.3 (9.3-fold) and 7.8 ± 0.6 (19.5-fold) mg/dL. BUN of the synbiotics-treated CKD group was 88.4 ± 6.7 (6.3-fold) and 124.9 ± 1.0 (7.8-fold) mg/dL compared with the control rats at the 3rd and 5th weeks, respectively. The serum creatinine of the synbiotics group were 2.1 ± 0.2 (7-fold) and 3.6 ± 0.4 (nine-fold) mg/dL. As shown in Table 3, BUN and creatinine were elevated less slowly in the synbiotics-treated group than the CKD group, indicating that adenine induces kidney damage, which can be slowed down by synbiotic treatment.

### 3.3. The Animal Model 2: Five-Week Adenine Diet Followed by 10-Week Synbiotic Treatment

**Figure 6:**
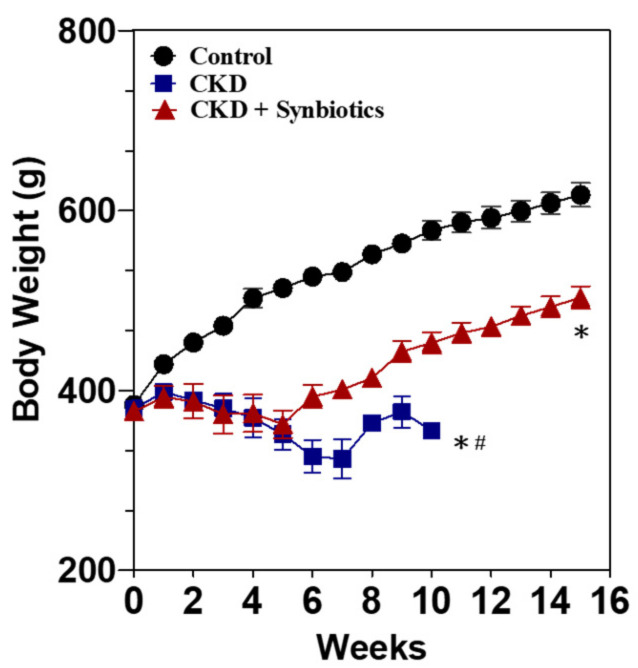
The body weight changes in animal model 2. The bodyweight of the controls (●), CKD rats (■), and synbiotics-treated CKD rats (▲). Adenine treatment causes animal death in the 10th week. A significant difference between the controls vs. CKD group vs. synbiotics-treated CKD group was analyzed using ANOVA. *, *p* < 0.05. A significant difference between the CKD group vs. the synbiotics-treated CKD group was analyzed using ANOVA. #, *p* < 0.05. *n* = 6 for each group. Abbreviation: CKD, chronic kidney disease.

The animal model 2 was categorized into three groups, including the control group, CKD group (oral gavage with 5-week adenine 250 mg/kg BW/day, followed by regular chow diet), and synbiotics-treated CKD group (fed with five-week adenine 250 mg/kg BW/day, followed by 10-week regular chow diet and oral gavage with synbiotics) (Figure 1). At the end of the 5th week, the CKD group and the synbiotics-treated CKD group lost about 30% of BW compared to the control rats (Figure 6). As shown in Table 4, the BUN levels of the CKD group and the synbiotics-treated group were significantly elevated at 207 ± 23 (13-fold) mg/dL, and the creatinine increased at 7.1 ± 1.0 (17.7-fold) compared with the wild-type rats at the 5th weeks. The results showed that the adenine diet successfully induces renal function damage, with poor appetite and BW loss, which mimic advanced CKD in humans. Adenine treatment causes animal death in the 10th week, as previously reported [22]. However, BW and kidney function could be rescued partially by the 10-week synbiotic treatment. The BUN levels of the synbiotics group were 77.9 ± 9.7 (5.2-fold) mg/dL, and the creatinine was 1.3 ± 0.2 (4.3-fold) mg/dL compared to the control rats at the 15th weeks (Table 4).

**Figure 7:**
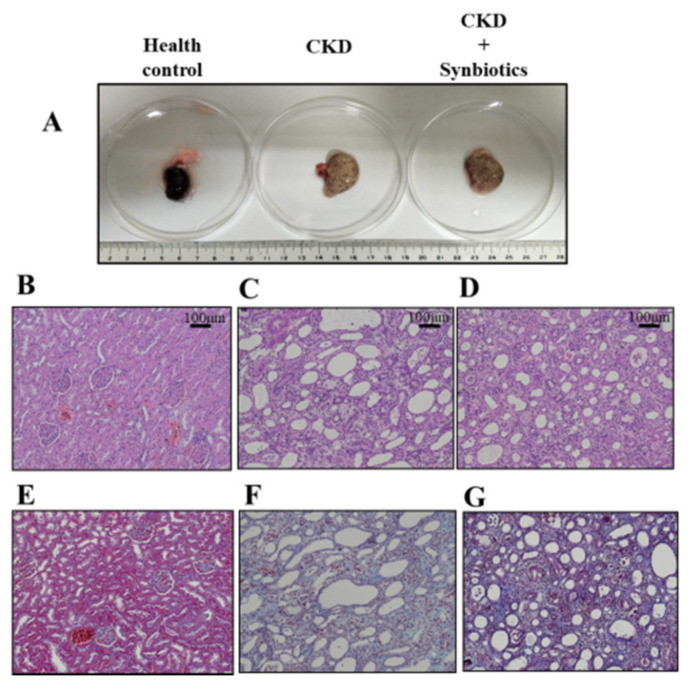
The kidney pathology in the controls, CKD rats, and synbiotics-treated CKD rats. (**A**) The gross examination of the kidney. (**B**–**D**) Hematoxylin and eosin stains of rat kidney tissues in control (**B**), CKD (**C**), and synbiotics-treated CKD (**D**). (**E**–**G**) Masson’s trichrome stains of rat tissues in control (**E**), CKD (**F**), and synbiotics-treated CKD (**G**). *n* = 6 for each group.

We also observed kidney pathology. As shown in Figure 7, the kidney fibrosis was rescued by synbiotic treatment. However, the mechanism of synbiotic treatment against renal fibrosis deserves further investigation. Collectively, we constructed an adenine-induced CKD rat model, and the synbiotic treatment may act as a treatment strategy for the CKD.

**Figure 8:**
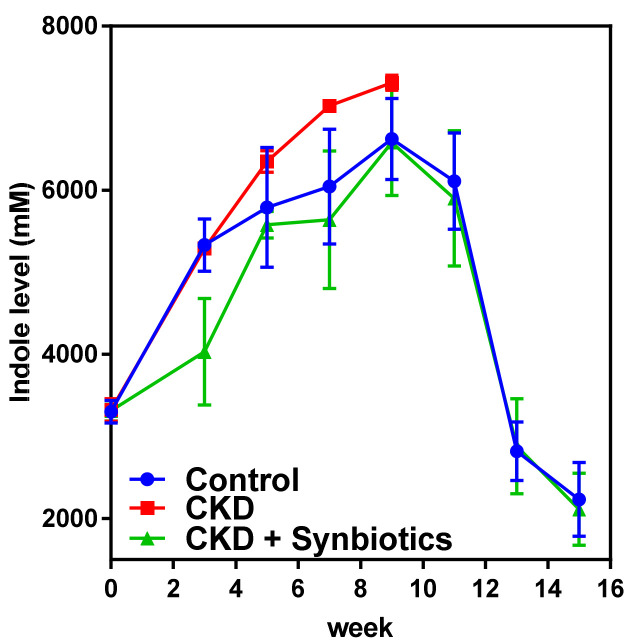
The time-series level of rat fecal indole in animal model 2. The fecal indole content in controls, CKD rats, and synbiotics-treated CKD rats. Adenine treatment causes animal death in the 10th week. *n* = 6 for each group.

As shown in Figure 8, CKD rats had a higher level of indole than controls. Adenine treatment causes animal death in the 10th week. Interestingly, the synbiotics-treated CKD rats had a lower level of indole than the CKD rats and control rats. These results suggest that synbiotic treatment may reduce fecal indole.

**Figure 9:**
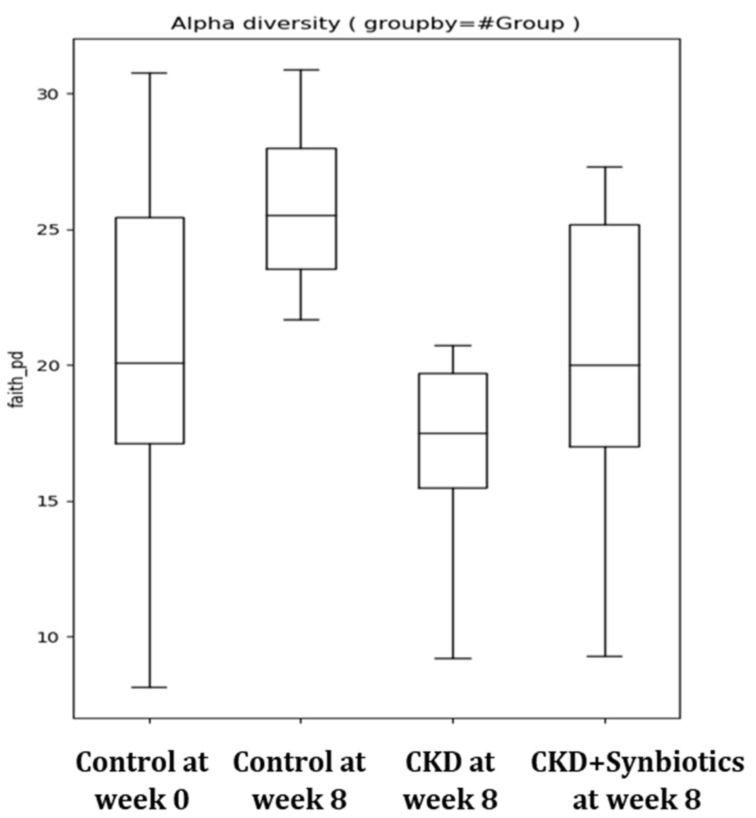
Gut microbiota diversity in the rat model (*n* = 6 for each group).

The CKD rat model showed increased fecal indole levels as well as the abundance of indole producing bacterium *Clostridium* up to 10%. However, the fecal indole level of CKD rats reduced while treated with synbiotics. Additionally, the gut indole-producing bacterium *Clostridium* was reduced to a normal distribution (Table 5). Furthermore, the results demonstrated that gut microbiota diversity was reduced in CKD rats but can be restored after synbiotic treatment (Figure 9).

## 4. Discussion

Our results demonstrated that in the adenine-induced CKD rat model, synbiotics ameliorated gut dysbiosis, reduced fecal indole amount, and slowed down the progression of CKD. This finding provides a promising therapeutic strategy to decelerate the progression of CKD. In ESKD patients, we found that fecal indole tends to be higher in the CKD patients as compared to the healthy controls, but the difference did not achieve statistical significance. However, the blood level of indoxyl sulfate was significantly higher than that of healthy controls. This implies that under an equivalent indole production rate, the impaired renal excretion contributes to the accumulation of this notorious uremic toxin. Although the abundance of indole-producing bacteria was not significantly different between ESKD patients and normal controls, two SCFA-producing bacteria, *Faecalibacterium* and *Roseburia*, were reduced in ESKD patients. This may contribute to gut dysbiosis. We also identified that three genera Fusobacterium, Shewanella, and Erwinia, were only found in the ESKD patients but not in the healthy controls.

Indoxyl sulfate is a protein-bound uremic toxin, whose excretion is reduced in CKD patients. Previous studies have proved that the gut microbiome is a potential source of uremic toxins. For example, indole or, more specifically, indoxyl sulfate is generated from the metabolism of tryptophan [34]. They are cleared by proximal tubules and increased in patients with CKD [35,36]. Nephrotoxic via OAT-mediated will be uptake by proximal tubule cells and activate nuclear factor (NF-κB) along with plasminogen activator inhibitor type I [37,38,39]. Another one is phenols: para (*p*)-cresol, the product of the breakdown of tyrosine and phenylalanine by intestinal bacteria. Phenols (*p*-cresol and phenol) and indole, the precursors of indoxyl sulfate, are nitrogenous metabolites produced by intestinal bacteria from tyrosine and tryptophan. In the beginning, we hypothesized that indole-producing bacteria are more abundant in CKD patients. However, the results did not show a significant difference in indole-producing bacteria between healthy controls and CKD patients. Nevertheless, the blood level of indoxyl sulfate and *p*-cresol sulfate are significantly higher in the CKD patients, indicating that it is the impaired excretion that majorly contributes to the circulating level difference. However, from our rat data, we can see that synbiotics significantly reduce fecal indole content and indole-producing bacteria. This finding provides evidence that though the basal composition of indole-producing bacteria is similar between healthy control and CKD patients, synbiotic treatment may further change the gut microbiota and reduces the source of these gut microbe-derived uremic toxins.

A healthy human body harbors various commensal microorganisms, which have coevolved with human beings in a symbiotic relationship. Most of them inhabit the gastrointestinal tract. Over 50 bacteria phyla are known, but generally, only 6–10 phyla are found in the human gut. Their abundance, relative proportions, and the number of species make up the criteria for “core microbiota.” Common microbiota includes *Bacteroidetes, Firmicutes, Proteobacteria, Actinobacteria, Verrucomicrobria*, and *Fusobacteria*. Although the predominant phyla are *Bacteroidetes* and *Firmicutes*, quite a high variation exists at the levels of genera, species, and strains. Knowledge of the relationship between gut microbiota and health/disease is rapidly growing. Results indicate the importance of a balance of microbial groups in the gut. However, it should be noted that most of the study populations are westerners. Furthermore, findings from the Human Microbiome Project and our previous study show that each individual’s microbiome is unique [40,41]. Recent understanding of the composition and metabolism of the human microbiota shows that it has an important influence on human health. 16S rRNA sequencing offers an affordable way to profile microbiota, and interactions between different bacterial communities and their environments can be comprehensively analyzed by metagenomics research. Moreover, some bacteria that are strongly associated with specific diseases were thought to be biomarkers [42,43]. The use of the stool as diagnostic targets may avoid unnecessary biopsies; it offers an inexpensive, non-invasive, and easily accessible early detection and prognosis tool [13,44,45,46]. Based on our indole results, the modified Kovács analysis can analyze the fecal indole. This novel methodology provides an efficient diagnosis method that might be used to detect the progression of CKD in the future.

According to previous studies, CKD patients possess different gut microbiome profiles comparing to healthy controls. The abundance of *Acinetobacter*, *Lactobacillus*, *Lachnospira,* and *Ruminococcus gnavus* was reported to be capable of differentiating CKD patients from healthy controls [47,48]. We are interested in whether the overall bacterial composition profiles from our pilot dataset can provide such kinds of biomarkers between CKD patients and controls. When comparing the top 10 most abundant microbial genus in two groups (Table 5), we found the percentages of three genera, *Streptococcus, Parabacteroides,* and *Dorea,* increased in CKD patients. Conversely, the relative abundance of *Faecalibacterium*, *Prevotella*, *Clostridium,* and *Roseburia* was found slightly decreased in CKD patients. Among them, *Faecalibacterium* and *Roseburia* produce SCFA and are reduced in ESKD patients, consistent with SCFA insufficiency that is observed in gut dysbiosis [49,50,51,52]. Of note, we did not assess other factors affecting the blood level of indoxyl sulfate, most notably the function of the gut-blood barrier, which has been reported to be disturbed in CKD subjects [53].

## 5. Conclusions

Collectively, our observational study identified the gut microbiota and fecal indole difference between CKD patients and healthy subjects. The interventional study in the CKD rat model demonstrated that gut dysbiosis and renal function impairment could be ameliorated by synbiotic treatment. This is evident from the altered microbiota composition and improved renal function. However, in this study, we did not examine the effect of antibiotic therapy or microbiota transfer, which deserves further investigation. This alteration in microbiota composition and improvement in renal function acknowledges that synbiotics might be a promising strategy for CKD patients.

## Figures and Tables

**Table 1:** Demographic characteristics of human study participants.

	Healthy Controls	ESKD Patients	Total
Patient number	22	40	62
Age (year)	62.3 ± 10.0	69.5 ± 13.2	68.6 ± 13.0
Male gender (%)	50	50	50

**Table 2:** Top 10 abundant fecal microbes in healthy controls and ESKD patients.

Healthy Controls		CKD Patients	
Top 10 Genus	Percentage	Top 10 Genus	Percentage
*Bacteroides*	50.85%	*Bacteroides*	49.15%
*Prevotella*	7.51%	*Prevotella*	5.54%
*Clostridium*	4.38%	*Clostridium*	3.76%
*Faecalibacterium*	3.96%	***Streptococcus***	**3.45%**
*Roseburia*	2.89%	***Alistipes***	**3.29%**
*Ruminococcus*	2.78%	***Parabacteroides***	**3.12%**
*Alistipes*	2.73%	***Succinispira***	**3.05%**
*Megamonas*	2.43%	*Ruminococcus*	2.94%
*Succinispira*	2.30%	*Faecalibacterium*	2.73%
*Arcobacter*	2.21%	***Dorea***	**2.62%**

**Table 3:** The blood test of animal model 1. The blood urea nitrogen (BUN) and serum creatinine changes in the controls, CKD rats, and synbiotics-treated CKD rats. All data were presented as means ± SEM. A significant difference between the controls vs. CKD group vs. synbiotics-treated CKD group was analyzed using ANOVA. *, *p* < 0.05. *n* = 6 for each group. A significant difference between the CKD group vs. the synbiotics-treated CKD group was analyzed using ANOVA. #, *p* < 0.05. Abbreviation: CKD, chronic kidney disease.

Animal Model 1				
Serum Test	Week	Control	CKD	CKD + Synbiotics
Blood urea nitrogen (mg/dL)	0	14.2 ± 1.3	15.4 ± 0.7	15.2 ± 0.4
	3	14.1 ± 1.5	108.5 ± 0.9 *	88.4 ± 6.7 *^,#^
	5	15.9 ± 0.6	214.0 ± 20.0 *	124.9 ± 1.0 *^,#^
Serum creatinine (mg/dL)	0	0.2 ± 0.0	0.2 ± 0.1	0.3 ± 0.2
	3	0.3 ± 0.1	2.8 ± 0.3 *	2.1 ± 0.2
	5	0.4 ± 0.1	7.8 ± 0.6 *	3.6 ± 0.4 *^,#^

**Table 4:** The blood test of animal model 2. Adenine treatment causes animal death in the 10th week. All data were presented as means ± SEM. A significant difference between the controls vs. CKD group vs. synbiotics-treated CKD group was analyzed using ANOVA. *, *p* < 0.05. *n* = 6 for each group. A significant difference between the CKD group vs. the synbiotics-treated CKD group was analyzed using ANOVA. #, *p* < 0.05. Abbreviation: CKD, chronic kidney disease.

Animal Model 2				
Serum Test	Week	Control	CKD	CKD + Synbiotics
Blood urea nitrogen (mg/dL)	0	15.8 ± 0.2	15.1 ± 0.4	15.7 ± 0.4
	3	16.0 ± 1.0	110.1 ± 11.0 *	109.7 ± 7.4 *
	5	17.8 ± 0.6	214.2 ± 21.8 *	201.2 ± 26.6 *
	7	17.8 ± 0.8	191.2 ± 20.4 *	100.2 ± 13.4 *^,#^
	9	16.0 ± 0.5	190.4 ± 0 *	87.0 ± 11.2 *^#^
	11	17.4 ± 1.0	-	83.3 ± 5.9 *
	13	15.6 ± 0.9	-	80.0 ± 6.9 *
	15	14.9 ± 1.0	-	77.3 ± 9.7 *
Serum creatinine (mg/dL)	0	0.2 ± 0	0.2 ± 0	0.3 ± 0.1
	3	0.3 ± 0.1	2.2 ± 0.5 *	2.4 ± 0.3 *
	5	0.3 ± 0.1	7.1 ± 0.8 *	7.3 ± 1.6 *
	7	0.3 ± 0.1	5.5 ± 1.5 *	3.3 ± 0.5 *^,#^
	9	0.3 ± 0.1	4.2 ± 0 *	1.7 ± 0.3 *^,#^
	11	0.3 ± 0.1	-	1.4 ± 0.1
	13	0.3 ± 0.1	-	1.3 ± 0.2
	15	0.3 ± 0	-	1.3 ± 0.2

**Table 5:** Composition of gut microbiota of controls, CKD rats, and synbiotics-treated CKD rats (*n* = 6 for each group).

Control		CKD		CKD + Synbiotics	
Top 10 Genus		Top 10 Genus		Top 10 Genus	
*Ruminococcus*	20.26%	*Parabacteroides*	19.52%	*Parabacteroides*	18.36%
*Parabacteroides*	16.16%	***Clostridium***	**19.22%**	***Lactobacillus***	**16.70%**
*Lactobacillus*	15.50%	*Ruminococcus*	17.07%	*Ruminococcus*	12.29%
*Clostridium*	10.07%	*Lactobacillus*	11.27%	***Clostridium***	**10.84%**
*Marvinbryantia*	9.75%	***Akkermansia***	**8.11%**	***Allobaculum***	**9.60%**
*Prevotella*	8.37%	***Anaeroplasma***	**5.86%**	*Alistipes*	8.44%
*Alistipes*	4.31%	*Marvinbryantia*	4.14%	*Anaeroplasma*	8.23%
*Sporobacter*	4.24%	*Alistipes*	4.00%	***Bifidobacterium***	**6.14%**
*Adlercreutzia*	2.15%	*Sporobacter*	1.62%	*Marvinbryantia*	3.56%
*Bifidobacterium*	1.54%	*Bifidobacterium*	1.13%	***rc4-4***	**1.71%**

## Data Availability

All data generated or analyzed during this study are included in this published article.
